# Reference as an Interactive Achievement: Sequential and Longitudinal Analyses of Labeling Interactions in Shared Book Reading and Free Play

**DOI:** 10.3389/fpsyg.2017.00139

**Published:** 2017-02-14

**Authors:** Vivien Heller, Katharina J. Rohlfing

**Affiliations:** ^1^Department of German Studies, School of Humanities and Cultural Studies, University of WuppertalWuppertal, Germany; ^2^Faculty of Arts and Humanities, Paderborn UniversityPaderborn, Germany

**Keywords:** reference, sequential organization, conditional relevance, observability, coordination, interaction, language acquisition, joint attention

## Abstract

The present study examines how young children and their caregivers establish reference by jointly developing stable patterns of bodily, perceptual, and interactive coordination. Our longitudinal investigation focuses on two mother–child dyads engaged in picture-book reading and play. The dyads were videotaped at home once every 6 weeks while the children aged from 9 to 24 months. Inspired by conversation analysis and multimodal analysis, our developmental approach builds on the insight that the situated and embodied production of reference is fundamentally an interactive achievement. To examine the acquisition of reference, we developed a descriptive instrument that takes account of not only the dyad's joint accomplishment but also each participant's contributions to it. The instrument is based on the sequential reconstruction of the jobs that both participants have to accomplish jointly in order to achieve reference: establishing visual perception as a relevant resource, constituting a domain of scrutiny, locating a target, and construing the (meaning of the) referent. Methodologically, these jobs serve as a *tertium comparationis* for the longitudinal comparison of both the adult's as well as the child's contributions to establishing reference. We used this instrument to examine (1) what bodily and verbal resources the participants employed, and (2) how their contributions to accomplishing the jobs changed over time. Findings showed that the acquisition of reference was closely related to the child's increasing ability to recognize, fulfill, and set up conditional relevancies. We conclude that the adult's dynamic and contextualized use of conditional relevancies, recipient design, and observability is a crucial driving force in the acquisition of reference.

## Introduction

Determining how young children come to understand that words refer to something has been a continuous topic in language acquisition research. For Bruner (1976, p. 69), the acquisition of reference entails the problem of “how one individual manages to get another to share, attend to, zero in upon a topic that is occupying him.” Arriving at a shared understanding of a referent is a substantial challenge when reference is conceived merely as words being mapped onto their referents, because in the real world, there are simply too many options when it comes to selecting one of the numerous potential referents (Trueswell et al., 2016). Considering the fact that speakers often produce “proxy” or “dummy” noun phrases (e.g., “what's-his-name”) for the referent, Clark and Wilkes-Gibbs (1986) asked how it is possible for participants to be sufficiently sure of having achieved a mutual understanding of the referent—a problem that Clark and Marshall (1981) referred to as the “mutual knowledge paradox.” This paradox also exists when reference is established non-verbally by, for example, pointing to an object within the coparticipants' joint perceptual space. Pointing is usually understood as a “communicative body movement that projects a vector from a body part” and “indicates a certain direction, location, or object” (Kita, 2003, p. 1). At first sight, the meaning of pointing seems to be self-evident in that it requires only the recipient to “trace, by symbolic extrapolation, a path from the gesture to the thing” (Fillmore, 1997, p. 6). Yet the mutual knowledge paradox remains, because pointing gestures only roughly indicate a certain area that may be populated by various persons, objects, and so forth. Even if the recipient manages to locate the pointed-to target and thus to resolve this perceptual ambiguity, she or he still needs to sort out another problem: Does the pointing refer to the object as such, or to one of its features; or does it simply predicate that the object is located in a particular area (see Kita, 2003, p. 3)? The meaning of the pointed-to target—the actual referent—still remains ambiguous. And yet, in everyday interaction, reference is usually achieved without problems.

In this article, we assume that participants themselves have developed procedural and linguistic solutions for dealing with perceptual and semantic ambiguities. Acquiring reference would then mean acquiring these procedural and linguistic solutions. Following a pragmatic perspective (Rohlfing et al., 2016), we assume that for a situation to become “shared,” interactants have to arrive at a joint understanding of the purpose of their activity. As a result, children need to learn “as much about the rules of dialogue” as they learn about the “lexical labels” (Bruner, 1976, p. 74).

A number of answers have been proposed in response to the question when and how children engage in establishing joint reference. In the following, we shall give a rough overview of relevant streams of research, and show how existing studies have mapped out the necessary cognitive and communicative resources as well as the necessary external resources for the acquisition of reference.

### Cognitive and communicative resources for establishing reference

Children have been found to engage in joint attention (JA) from 9 months onward. JA is achieved when both partners manage to engage with the same referent. However, it was results reported by Baldwin (1991, 1993) that first motivated a closer investigation of the child's sociocognitive abilities. She demonstrated that infants “are not just passive in the joint reference enterprise” (Baldwin, 1993, p. 398). They have a range of communicative means at their disposal with which not only to display their interest in objects, persons, and so forth but also to direct their coparticipant's attention (e.g., Liszkowski et al., 2004; Liszkowski, 2005; Begus and Southgate, 2012). They use these resources for both imperative and declarative purposes (Bates et al., 1976; Franco and Butterworth, 1996; Liszkowski et al., 2004, 2007). Moreover, they understand that their actions have a bearing on their partner, and they use this knowledge to elicit a label or further talk (Begus and Southgate, 2012; Begus et al., 2014). Pointing is among the first communicative means for directing the coparticipant's attention to objects and events (Bruner, 1983; Franco and Butterworth, 1991; Marcos, 1991; Butterworth and Itakura, 2000; Behne et al., 2012). At around 14 months of age, children accompany their pointing with the local deictic “da!” or “there” (Clark, 1978; Clark and Sengul, 1978; Murphy, 1978). Clark (1978) has proposed four stages in the development from deictic gestures to deictic words:

At the first stage, children use gestures like pointing to pick out an object for their “listeners.” At the second, they add to their gesture their first deictic word, often in the form *eh* (from adult *there*) or *da* (from adult *that*). Later still, at a third stage, they combine a deictic word with other words to form longer utterances like *That shoe*… Finally, at a fourth stage, they learn how to use deictic words in utterances without any accompanying gesture (p. 96).

Whereas the stages capture a progression in the child's use of deictic means, they do not reflect the need for deixis to also be embedded in the ongoing interaction. Yet to be successful, the child has to make sure that the partner is ready to perceive the pointing (“visual checking,” see Franco and Butterworth, 1996). In other words, pointing must be prepared interactively. Likewise, pointing grants relevance to a certain reaction by the recipient. Filipi (2013, p. 145) has shown that children first learn to establish joint attention and are then held “accountable for ‘doing’ something with that attention when it is provided.” Hence, it seems that the “recognition of a situation as communication” (Gliga and Csibra, 2009, p. 352) and the child's sensitivity to the organization and the purpose of the task is important for acquiring reference. Studies applying sequential analyses to young children's interactions stress the public nature or “observability” of each participant's actions as a crucial resource (Wootton, 1997; Kidwell and Zimmerman, 2006, 2007). What is lacking, however, is studies on early interactions showing how this “observability” is achieved and adapted to children's communicative and cognitive abilities.

### External resources for the acquisition of reference

Input-oriented approaches have examined how adults facilitate JA; how they modify their talk in episodes of JA; and how adult feedback affects developments in referential communication (see Ateş-Şen and Küntay, 2015, for an overview). Mothers have been found to point and refer to objects verbally more often in episodes of JA (e.g., Bruner, 1981; Tomasello and Farrar, 1986; Marcos, 1991). Furthermore, parameters for “referential transparency” (Trueswell et al., 2016, p. 11; Schmidt, 1996) have been identified that help children to attend to novel objects visually and thus to resolve ambiguities when linking objects with words (Pruden et al., 2006; Horst and Samuelson, 2008; Axelsson et al., 2012; Liszkowski, 2014; Trueswell et al., 2016; Yu and Smith, 2016). Adult coparticipants often present objects and actions in salient ways. They bring objects into the child's visual focus, shake them, and thus exploit the child's sensibility to human movement (e.g., Rader and Zukow-Goldring, 2010; Pitsch et al., 2014; Yu and Smith, 2016). In interactions with older children, mothers rely on verbal behavior to initiate and maintain their child's attention (Estigarribia and Clark, 2007). Although it could be shown that the caregiver's “input” in episodes of JA correlated positively with the child's use of pointing (Murphy, 1978; Marcos, 1991) and vocabulary (Tomasello and Farrar, 1986), these studies do not fully explain how participants actually arrive at a shared situation and a mutual understanding of the referent—a demand that goes clearly beyond joint attention to a particular target and requires the solving of semantic tasks.

Another strand of research investigating external resources looks beyond the phenomenon of JA. These studies take a broader view on the interactive contexts in which reference is established, and examine how interaction forms a source in the child's cognitive development (Vygotsky, 1998). A number of studies taking this approach have examined how the sequential structure of routines such as games or joint book readings is established (Ninio and Bruner, 1978; Snow and Goldfield, 1983; Filipi, 2009, 2013; Fantasia et al., 2014; Rossmanith et al., 2014; Heller and Rohlfing, 2015; Rohlfing et al., 2015, 2016). Based on a longitudinal study of one mother–child dyad, Ninio and Bruner (1978, p. 8) demonstrated that picture-book reading takes the form of a “standard action format” that consists of recurring dialogue cycles, each comprising an orderly sequence of moves. From a conversation analytic perspective, the structure is underpinned by “conditional relevancies” (Schegloff and Sacks, 1973); that is, normative expectations regarding what type of “relevant next” should follow a move of a certain type. In interactions with young children, adults have been found to “plan ahead” for conditional relevancies, thus guiding the child and creating “an interactional context that is most likely to occasion a desired response” (Mehus, 2011, p. 133). Such stable organization helps children to identify and predict recurring semantic-pragmatic elements in a sequence (Ratner and Bruner, 1978; Snow and Goldfield, 1983). Drawing on microanalyses, Rossmanith and colleagues have examined how caregivers structure book reading routines by shaping parts of activities into bigger or smaller dynamic “action arcs” with a beginning, build up, climax, and resolution (Rossmanith et al., 2014, p. 8). These render the structure of the routine visible for the child. By providing a recurring pattern, they facilitate the coordination of not only visible behaviors but also cognitive and perceptual operations (Rohlfing et al., 2016).

Focusing on *adult–adult interactions*, multimodal and sequential approaches have examined which “practical problems” participants have to solve when establishing reference. They have shown that joint reference is a sequentially organized process that requires participants' coordination of body posture, gaze, movements and verbal resources (Hanks, 2000; Hindmarsh and Heath, 2000; Goodwin, 2003b; Stukenbrock, 2009; Mondada, 2012; Sidnell and Enfield, 2016). The present study examines how *children* become involved in this interactive and sequentially organized process and how stable patterns of bodily, perceptual, and interactive coordination emerge over time. In the following section, we present an analytical instrument with which to describe this process. The instrument is based on the sequential reconstruction of the interactive jobs (see next section) that are constitutive for establishing reference. Using these jobs as a *tertium comparationis*, we examine how each job is achieved interactively at different data points and relate changes in the devices available to children and their shares in performing the jobs to changes in the adult's interactive demands and support. In the last section, we develop an explanatory account of what drives the acquisition of reference. We argue that fundamental features of interaction—sequential organization, recipient design, and observability—inform the supportive practices that adults employ to achieve joint reference in interactions with young children.

## A descriptive instrument for analyzing reference and its acquisition as interactive achievements

### Interactive jobs of establishing reference

When establishing reference, participants have to solve at least two problems: First, they have to deal with the *perceptual problem* of locating a target. Second, they have to solve the *semantic problem* of identifying or rather construing the referent. Hence, it appears that establishing reference inheres recurrent practical problems that require the ongoing and dynamic coordination of the participants' bodily and visual conduct. This is why participants rely on procedural solutions or “practical methods” (Garfinkel, 1967) that enable them to treat and perform “establishing reference” as an “unproblematic” activity in their everyday lives. Building on a framework based on sequential analyses of establishing reference in different settings such as dinner talk, guided tours, self-defense classes, physician–patient consultations (Stukenbrock, 2009, 2015), and picture-book reading (Heller and Rohlfing, 2015), we assume that the procedural solution to establishing reference entails four sequentially ordered jobs.

#### Job 1: establishing visual perception as a relevant resource

To make a pointing gesture perceptible, the pointing person has to establish her or his body as a perceptually relevant resource (Hindmarsh and Heath, 2000; Goodwin, 2003b; Stukenbrock, 2009; Mondada, 2012). Therefore, bodily displays must be coordinated with the recipient's visual attention. Hindmarsh and Heath (2000) have shown that speakers employ verbal resources such as deictic terms (“here!”) to highlight the very moment at which visual orientation becomes relevant—a resource that is also employed in interactions with children (Estigarribia and Clark, 2007, p. 804). The recipient, on the other hand, is required to direct her or his visual attention toward the speaker and to understand that the partner's arm or index finger is not relevant in itself but should be interpreted as an instrument referring to something else and thus serving as an intermediary locus of attention (Stukenbrock, 2009; Rader and Zukow-Goldring, 2010).

#### Job 2: constituting a domain of scrutiny

Next, the recipient needs to understand what space the speaker is orienting toward. It is important to emphasize that the speaker's display of attention—her or his orientation toward a certain space by posture, pointing, or local deictics—does not yet indicate a particular object in space. Rather than transparently locating the target itself, it “specifies…a domain of scrutiny, a region where the addressee should begin to search for something that might count as target” (Goodwin, 2003a, p. 73). The co-participant is thus required to reorient her or his visual attention; that is, to shift it from the body of the speaker to a “search space” (Stukenbrock, 2009, p. 304). At the same time, the speaker needs to monitor whether the co-participant construes the search space in the same way as her- or him self. Hence, this job is accomplished when both participants have established a particular space as a shared focus of attention.

#### Job 3: locating the target

This job requires the recipient to determine the particular target of the pointing gesture. Unlike Butterworth, we do not assume that the act of locating coincides with the identification of the referent. Butterworth (2003) suggests that certain ecological mechanisms enable a “‘meeting of minds’ in the selfsame object” (p. 22). Likewise, other studies have assumed that locating a target already implies understanding its meaning (e.g., Pruden et al., 2006; Axelsson et al., 2012; Trueswell et al., 2016). Admittedly, locating the target and construing the referent are often achieved at one go. Yet, misunderstandings and repairs do occur in the process of establishing reference (see below), suggesting that locating a target and construing the referent are in fact different achievements (Stukenbrock, 2009, 2015). Whereas locating a target requires a perceptual effort (which may lead to shared perception), construing the referent is a semantic process (occasioning shared understanding). Our own analyses of the ways in which not yet competent members are involved in establishing reference (Heller and Rohlfing, 2015) provide further evidence for the need to distinguish between the two.

#### Job 4: construing the referent

Once the target is located, the recipient needs to disambiguate its meaning. Therefore, she or he needs to tie acts of pointing or verbal deictics and labels “to the construals of entities and events provided by other meaning-making resources as participants work to carry out courses of collaborative action with each other” (Goodwin, 2003b, p. 218). Hence, to identify the referent, the coparticipant draws on contextual resources; that is, her or his understanding of the joint activity (e.g., book reading, building a tower) in which the reference is embedded (Hindmarsh and Heath, 2000; Liszkowski, 2014). She or he then develops hypotheses about the meaning of the pointed-to target (Stukenbrock, 2009, p. 307). This semantic work is conducted visibly and verbally: Adult recipients often display their understanding that can then be confirmed, specified, or repaired by the speaker (Stukenbrock, 2015, p. 316).

To summarize, we conceptualize reference as an interactive and sequentially organized process that requires participants to observably and methodically orient themselves toward four jobs. Whereas previous developmental research has focused mainly on Jobs 1 and 3 (Estigarribia and Clark, 2007), sequential analyses provide evidence that establishing reference also requires participants to constitute a domain of scrutiny and to construe the referent. The four sequentially ordered jobs thus serve as a procedural solution to practical problems of perceptual and semantic ambiguity. Note that scope of our descriptive instrument covers basic forms of reference; that is, activities in which participants refer to something in their immediate surroundings. It does not apply to references to past, future, or fictitious events.

### Descriptive levels of the instrument

Starting from the perspective that reference is fundamentally an interactive achievement, a developmental approach to reference has to tackle the question how *individual* abilities can be described without ignoring the fact that reference is a *collaboratively* organized process. Our solution to this problem is to view the interactive process itself as a part of the analysis. Therefore, we build on an analytical approach developed by Hausendorf and Quasthoff (2005) designed originally to examine the acquisition of narrative competence. Adopting this instrument for the acquisition of reference, we distinguish two levels of description: the level of *jobs* and the level of the *devices* needed to get the jobs done.

*Jobs* represent the organizational tasks (Sacks, 1995; Quasthoff et al., 2017) the participants orient toward in the joint achievement of reference. Because these jobs follow a sequential logic, this level of description captures the sequential organization of reference. Furthermore, the present analysis will demonstrate that each of the four jobs is organized as a two-part exchange or adjacency pair in which a move of type A establishes a “conditional relevance” for a move of type B (Schegloff and Sacks, 1973). Hence, the second move is functionally dependent on (or made normatively expectable by) the first. Each job has been achieved when the second pair part of the expected type has been produced. Reference, then, is successfully established when each of the four jobs has been fulfilled regardless of how and by whom. The jobs thus serve as a *tertium comparationis* for the longitudinal comparison of both the adult's and the child's contributions to establishing reference.

*Devices* is the term given to the bodily, prosodic, and verbal means or resources with which the jobs are accomplished. They describe each participant's contributions to the jobs. Moreover, different devices can be deployed to accomplish the jobs.

By distinguishing between interactive jobs and devices, the instrument takes into account both the dyad's joint accomplishment and each participant's contributions to establishing reference. It thus provides the basis for a longitudinal comparison of the adult's and the child's contributions without losing sight of the fact that reference is coconstructed. This allows us to examine (1) what bodily-visual and verbal resources participants employ to accomplish the jobs and (2) how their shares in the jobs change over time.

## Materials and methods

### Participants

The longitudinal analysis is based on video recordings of face-to-face interactions between caregivers and two typically developing children as they aged from 9 to 24 months. These dyads were selected from a larger corpus (e.g., Rohlfing et al., 2015) and include children of both genders. Based on our corpus, they represent “typical” courses of language acquisition. Participants were recruited in the German city of Bielefeld and its surroundings. The mothers' educational background was comparable; both had university degrees.

### Data collection and transcription

Each family was visited at home once every 6 weeks (12 data points). Two different activities were videotaped, free play (lasting 20–25 min) and picture-book reading (lasting 5–10 min). For the latter activity, the dyads were given a colorful folder: Each page presented photographs showing, for example, a spoon on a mug or a child on a swing. Altogether, the corpus comprises 10.5 h of video recordings. For each point of data collection, three to eight episodes were transcribed in Elan (EUDICO Linguistic Annotator; Lausberg and Sloetjes, 2009). The 93 transcripts cover 42 min of interaction. The transcription follows the notation conventions of Gesprächsanalytisches Transkriptionssystem 2 (GAT 2, Couper-Kuhlen and Barth-Weingarten, 2011). It depicts participants' verbal, non-verbal (e.g., pointings, depictive gestures, gaze), and paraverbal actions (e.g., accentuation, pitch movement, loudness) in their sequential order (see Appendix). All transcripts were checked by two research assistants. Parents provided written informed consent for the study as well as specific consent for the publication of images in the transcripts. The names used in the transcripts are pseudonyms. The first number in the transcript title refers to the dyad (01 and 07); “BR” and “FP” refer to “book reading” and “free play.”

### Analytical procedure

The analysis entailed two steps: Drawing on conversation analysis (Sacks, 1995) and multimodal analysis (Streeck et al., 2011), we first examined how each job was achieved by the dyad in different interaction episodes (section Age-Related Sequential Analyses). This sequential analysis focused on the devices adults and children employed to get the jobs done. Examples are presented for four age spans (9–14, 15–17, 18–22, and 23–24 months). The age spans were not determined *a priori*, but are based on our analyses. They reflect changes in the adults' interactive demands and/or the children's contributions to establishing reference. In the second step, we related changes in the children's devices and shares in the jobs to changes in the adult's interactive demands and support (sections Longitudinal Comparison: Children's Devices and Shares in the Jobs and Longitudinal Comparison: Adults' Devices and Shares in the Jobs).

## Analyses and findings

### Age-related sequential analyses

#### Establishing visual perception as a relevant resource (Job 1)

##### 9–14 months

**Figure 1:**
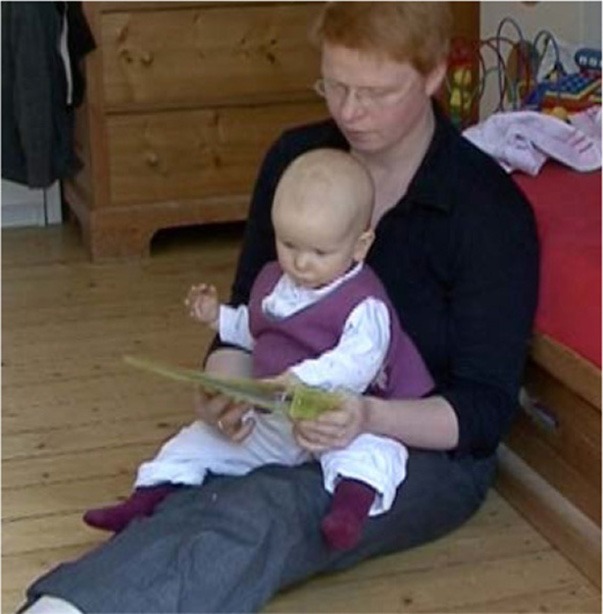
**Nested arrangement**.

**Figure 2:**
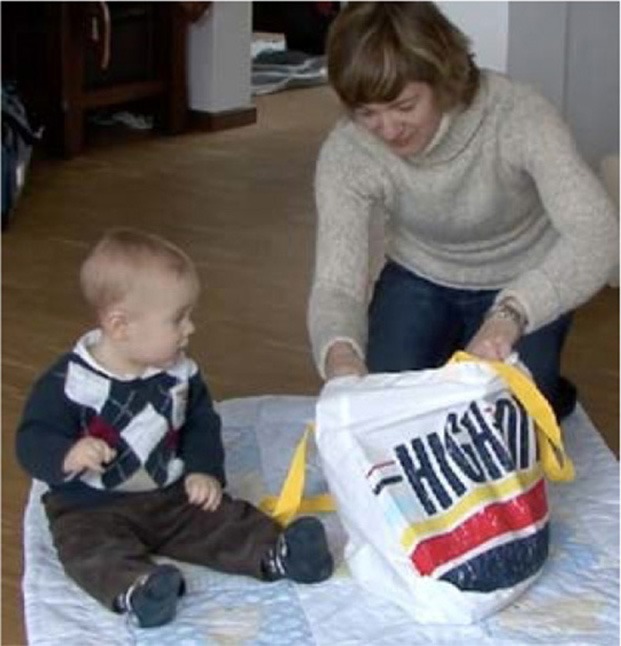
**Side by side**.

How visual perception is established as a relevant resource depends decisively on the participants' bodily arrangements. For book reading with young children, mothers typically arrange a nested configuration (Ochs et al., 2005) and position the child on their lap facing outwards (Figure 1). Thus, the child shares a visual field with the mother and does not need to redirect her or his gaze from the mother's body to the pointed-to domain of scrutiny (Job 2). When the mother points to the book, both her finger and the domain of scrutiny can be perceived simultaneously (see Yu and Smith, 2013). During play, participants sit face to face or side by side (Figure 2). This arrangement requires the pointing person to first draw the coparticipant's visual attention to her or his own body.

In the first sequence, Lea (9 months) is in a nested position.

**Table:** 

(1) 07-BR-spoon (9 months)							
001	L	[((turns page, looks at rings))]					
**002**	M	**[AH::::**	**was**	**ham**	**wir**	**denn**	**DA:::;]**
		AH:::	what	do	we	have	the::re;
003	L	((looks at picture))					

At the beginning of episode (1), the participants' visual attention is not coordinated. While Lea is turning the page and looking at the rings of the file, the adult is looking at the picture. At this moment, the adult produces a what question that is prefaced with a lengthened interjection (line 2: “AH::::”). The question has a standard format:

**Table:** 

(2) 07-BR-spoon (9 months)								
**002**	**M**	**[AH::::**	**was**	**ham**	**wir**	**denn**	**DA:::;]**	
		AH:::	what	do	we	have	the::re;	
(3) 01-BR-book (10 months)								
**006**	**M**	**OAH**	**(.)**	**was**	**ham_wa**		**denn (-)**	**↑DA::;**
		OAH	(.)	what	do	we	have	THE::RE;

In both examples, the interjection serves as an audible display of the speaker's excitement about having discovered something new. The pronoun “we” indicates that the speaker addresses the question to both herself and the coparticipant, thus making *joint* attention relevant. The local adverb “da”/“there” is lengthened and accented (see Estigarribia and Clark, 2007). Even if the child cannot understand the lexical meaning of the words, the prosody is designed to arouse her or his attention (see Pitsch et al., 2014, for a similar finding). Thus, in this sequential position, the what question does not ask for a label but establishes a sequential implication for the child to direct her or his gaze toward the mother's body (in this case: her hand). The what question and the bodily response thus form an adjacency pair; that is, a two-part exchange in which the second move is functionally dependent on the first. Forming the first pair part, the what question sets up a conditional relevance for visual coordination as a second pair part. In our data, the children frequently treat the what question as sequentially implicative by redirecting their gaze toward the mother's hand in front of the picture.

In play situations, mothers place the object in front of the child and thus reduce the need for the child to shift her or his gaze between mother and object.

**Table:** 

(4) 01-FP-bag (10 months)				
001	M:	|KOMM	her	ole; |
		COME	here	ole;
002		**|**°**hhh**	**SCHAU**	**mal**.** |**
		°**hhh**	**LOOK**.	
		**|((opens bag))|**		
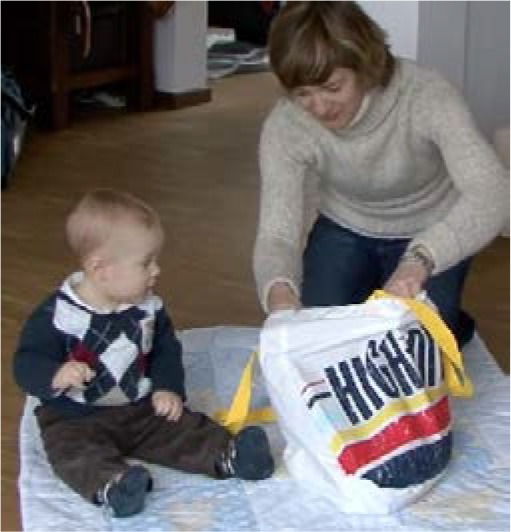				

When opening the bag, the mother publically displays her own attention through a sharp intake of breath (line 2; see Rossmanith et al., 2014). This is followed by the summons “LOOK.” (see also Murphy, 1978; Estigarribia and Clark, 2007; Pitsch et al., 2014; Rossmanith et al., 2014). Just like the what question, the summons forms a first pair part that establishes a conditional relevance for visual coordination.

##### 15–17 months

From 15 to 17 months onward, a variation in the division of labor can be observed. Every now and then, it is the child who initiates the job of establishing visual perception as a relevant resource, thus reversing the sequential obligations. In extracts (5) and (6), Lea attracts her mother's attention by displaying her own excitement.

**Table:** 

(5) 07-BR-red flower (15 months)			
001	L	((turns page))
**002**		°**h-**
003	M	BLUmen;
		FLOwers;
(6) 07-BR-mug (17 months)		
001	M	|U:::ND, |
		A:::ND,
		|((turns page))|
**002**	**L**	**oh;**
003		((rIF points to book))
004	M	ein LÖFfel,
		a SPOON,

To establish visual perception as a relevant resource, Lea employs devices used previously by the adult: breathing in (Excerpt 5) and, a few weeks later, interjections (Excerpt 6). Here, the child also points to the book (line 3), thus already initiating the next job.

##### 18–22 months

In this age span, another change could be observed in the book-reading situation. Now, the first job was sometimes skipped. Visual perception was made relevant only at the very opening of the book-reading routine. As soon as the routine got under way, neither child nor adult employed interjections, questions, or summons to display their own and elicit the coparticipant's visual attention. A decrease in verbal attention getters was also observed by Estigarribia and Clark (2007), albeit with respect to interactions with older children. In the following extract, Ole locates a target (by vocalizing and pointing) immediately after his mother has turned the page.

**Table:** 

(7) 01-BR-dino (19 months)					
001	M:	((turns page))		
**002**	**O:**	**|!Ä!O;**		**|**
		**|((points to tiger…)) |**			
		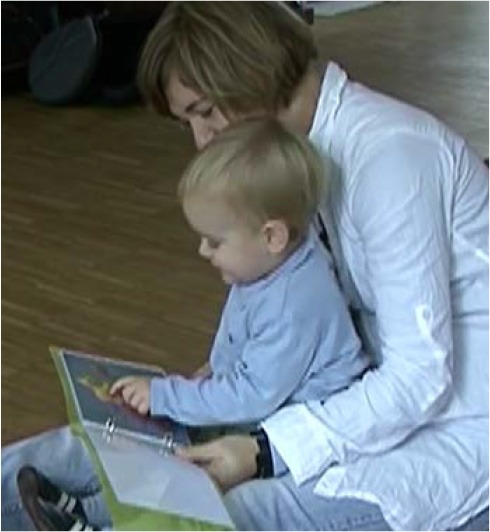			
003		|DAS-		|
		THAT-		
		|((…points to tiger))|			
004	M:	<<p>	was ist DAS;>		
			what is THAT;		

The skipping of the first job indicates that participants have arrived at a mutual understanding of the job and the overall activity—they mutually rely on each other's attention.

##### 23–24 months

From 23 months onward, it can be observed that children employ questions that the adult used months before. Given the fact that only a couple of weeks before, the coparticipants were found to mutually rely on each other's attention, this is surprising. The questions or prompts, however, are a device that enables the child not only to attract but also to direct the adult's attention in a more specific way (Clark, 1978) by, for example, asking for a label. The fact that the mother resists this obligation (as in Excerpt 8), reflects her heightened expectation with regard to Lea's ability to label the referent herself.

**Table:** 

(8) 07-BR-star (24 months)				
**001**	**L**	**|IST**	**das? |**
		**IS**	**that?**
		|points to picture|		
002	M	SAG_s	mir.
		TELL	me.

Table 1 summarizes the devices adults and children employ to establish visual perception as a relevant resource. The list is not meant to be exhaustive. In different spatial configurations participants might well-draw on additional resources.

**Table 1:** **Adults' and children's devices for establishing visual perception as a relevant resource**.

	**9–14 months**	**15–17 months**	**18–22 months**	**23–24 months**
Adult	Initiates job by setting up a relevance for visual coordination. Devices: Breathing in or interjection What question or summons		Higher expectation: Verbal cues are omitted	
Child	Responds by coordinating visual attention	Initiates job by setting up a relevance for visual coordination. Devices: Breathing in Interjections		Initiates job by setting up a relevance for visual coordination. Devices: What questions and summons

#### Constituting a domain of scrutiny (Job 2)

The most striking developments in constituting a domain of scrutiny can be observed between 9 and 14 months of age. In this period, the child comes to understand the book and the toy storage bag as domains of scrutiny. Again, this job is organized as an adjacency pair.

##### 9–14 months

To establish joint reference, adult and child need to constitute a domain of scrutiny in which the target can be located. This entails two demands: First, the child must come to understand *that* (and for what purpose) something should be searched for—a cognitive demand as formulated by Rohlfing et al. (2016). Second, the child must come to understand *where*—in which area—the search should be made. When the child is not in a nested configuration and does not “automatically” share the same visual focus with the mother, the adult frequently brings the domain of scrutiny into the child's immediate visual field.

**Table:** 

(9) 01-BR-dog (10 months)							
001	M:	GUCK mal;					
		LOOK;					
**002**		**|HIER;**	**|**				
		**HERE;**					
		**|((holds**	**book**	**above**	**Ole’s**	**head))**	**|**
003	O:	((touches				book))	
		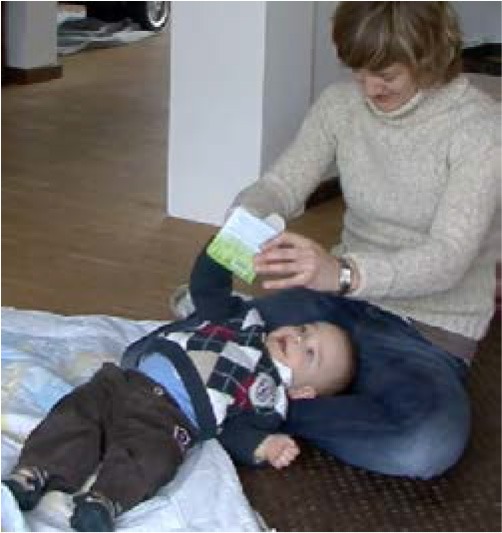					

In Excerpt (9), the mother holds the book above the child's head. Overlapping with this, she uses the local adverb “HERE;” as a device to instruct the child where and when to look. This summons forms a first pair part of another adjacency pair and establishes a conditional relevance; this time, for orienting toward the domain of scrutiny. Ole produces the expected second pair part by touching the book.

Another prototypical device is where questions. Like the summons, they set up a conditional relevance for orienting toward a search space. Yet in contrast to a summons, they entail two demands: first, the understanding *that* something should be searched for; and second, *what* this something is (Murphy, 1978). When constituting a domain of scrutiny, the adult makes only the first aspect relevant.

**Table:** 

(10) 07-BR-spoon (9 months)					
**004**	**M**	**|WO:**	**is**	**der**	**lÖffel; |**
		**WHE:RE**	**is**	**the**	**spoon;**
		**|((moves book, lifts it up)) |**			
005		WO::	ist	der	lÖffel;
		WHE::RE	is	the	spoon;
006	L	((touches	book	with	face))
007	M	WO	ist	der	lÖffel?
		WHERE	is	the	spoon?

Rather than conveying to the child *what* she is expected to search for, the mother's where question is designed to help Lea understand *that* she is expected to search for something. The accented “WHE::RE” (line 4) is designed to evoke a *searching stance* on the side of the child. Like the “HERE,” the where question projects the relevance of orienting toward the domain of scrutiny.

Overlapping with her question, the mother therefore marks the domain of scrutiny by moving the book and lifting it closer to Lea (line 4). This action indicates that the mother does not yet expect Lea to understand that the book itself, located right in front of Lea, constitutes the search space. Nonetheless, Lea does not produce a relevant next action. After being asked the question a second time (line 5), Lea bends forward and touches the book with her face (line 6). By repeating the question for a third time (line 7), the mother, however, does not ratify this reaction as an adequate response.

The analysis reveals that constituting a domain of scrutiny depends crucially on a mutual understanding of the current context of interaction. In this case, this job is not achieved because it requires the child to understand the purpose for which the book is being used. Although the domain of scrutiny is already in the child's visual focus, it is not recognized as such. This shows that constituting a domain of scrutiny is not only a matter of visual orientation but likewise a matter of *understanding the purpose of searching:* “Beyond the visual conduct, participants draw upon the activities in which reference emerges and forms a part, in order to produce, and make sense of, reference” (Hindmarsh and Heath, 2000, p. 1857).

In the context of book reading, understanding the purpose also involves knowing how to deal with pictures. During the first episodes of book reading, the child explores the book as an object:

**Table:** 

(11) 07-BR-pen (14 months)					
005	M	[WO	ist	der	stift; ]
		WHERE	is	the	pen;
006	L	[((tries	to	grasp	pen)) ]
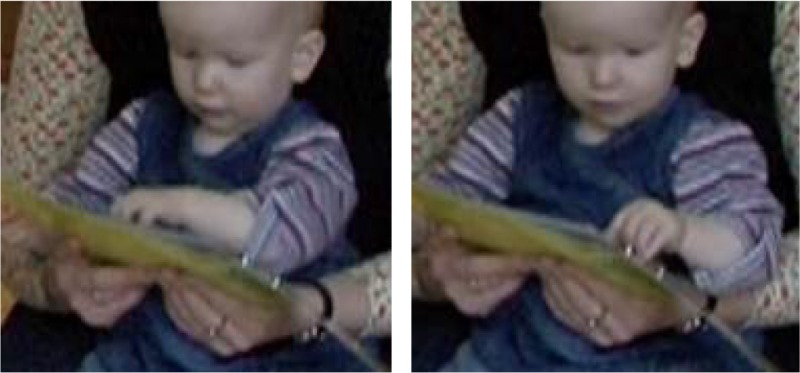					
007	M	ah	den	möchtste	wieder
		you	wanna	take	it
		GREIfen; = ne,			
		again; = right,			
008		= GEHT	nich;		
		doesn’t	work;		
009		|DA	is	der	stift. |
		THERE	is	the	pen.
	|((traces pen with rIF)) |				
((…))					
019	L	[((strokes with rIF over picture ]			
	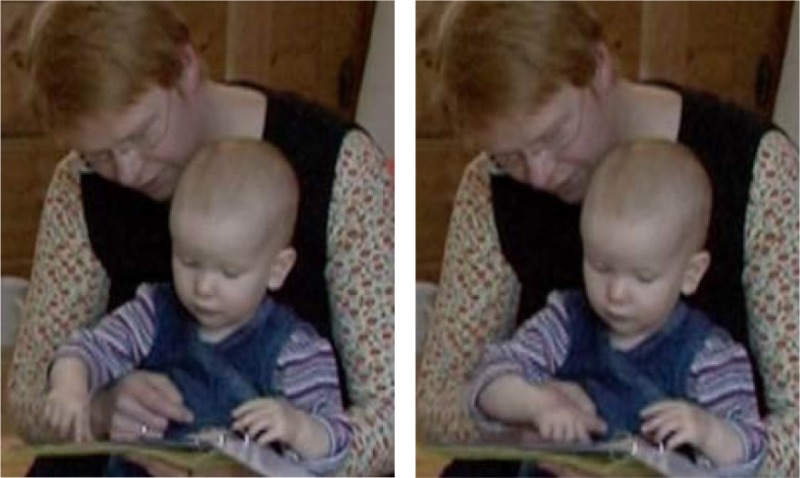				
020	M	[ja	is	ganz	GLATT; (-) ]
		yes	it’s	completely	SMOOTH;

Responding to the mother's where question (line 5), Lea grasps the rings of the file (line 6). The adult allows time for exploring the materiality of the pictures and thus for experiencing the physical impossibility of taking something “out of the book.” When locating objects herself, the mother *traces* their form (line 9), thus pointing to the depicted object and, at the same time, highlighting its depictive nature as such (“completely SMOOTH”; see Rohlfing et al., 2015, for similar strategies). Understanding depiction as such is a prerequisite for understanding what can be done with books and how they constitute a domain of scrutiny (see Ganea and Canfield, 2015, for a recent summary).

##### 15–17 months

From 15 months onward, children usually display an understanding of the book-reading routine. As soon as they know how the book is used, adults do not need to establish the book as a domain of scrutiny. Therefore, this job is skipped in this particular routine:

**Table:** 

(12) 07-BR-fishing rod (15 months)			
002	L	((turns page))	
003	M	!OH!;	
004		eine ANGel;	
		a fishing rod;	
005	L	[((lifts lh, [holds it))	]
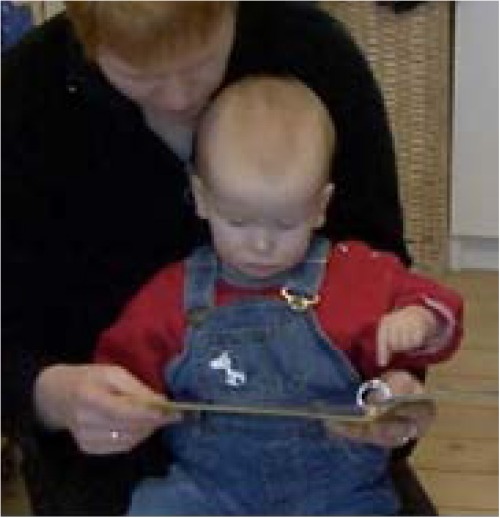			
006	M	[EIne ANGel;	]
		a fishing rod;	

Lea turns page and keeps her eyes on the book. The mother initiates the next cycle of establishing reference by displaying her excitement (Job 1). Then she immediately labels the referent (Job 4). Lea's hand is held in the air; it is not clear whether it depicts the fishing rod or is just held “ready.”

In the play setting, the job retains its importance. At 15–17 months, children start to use pointing to refer to distant entities that the co-participant is currently not oriented toward. In the following example, Ole establishes visual attention as a resource (Job 1) by standing up, moving into his mother's visual focus, and initiating eye contact. Then he points behind him (where a visitor is waiting behind the corner), thereby constituting a domain of scrutiny (Job 2).

**Table:** 

(13) 01-BR-thinking (17 months)					
021	O:	((stands up, moves into M’s visual focus))			
021		|!DA!-	|		
		!THERE!-			
		|((looks at M, points to person standing behind the wall))			|
		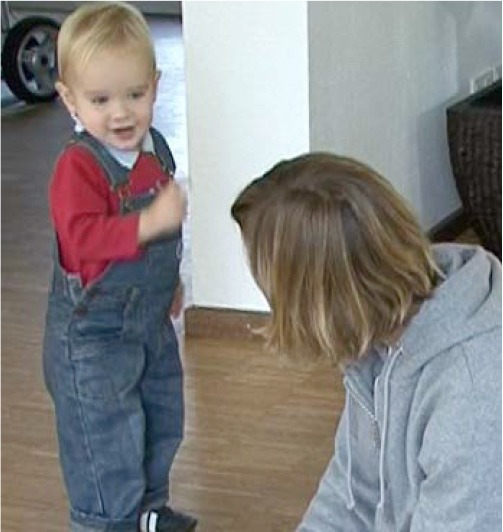			
022	M:	sanDAlen;			
		sandals			
023	O:	|!DA!-	|		
		!THERE!-			
		|((looks at M, points to place behind him)) |			
024	M:	wollts nochma GUCKen geh:n,			
		wanna go looking again,			
025	O:	((thinking face))			
		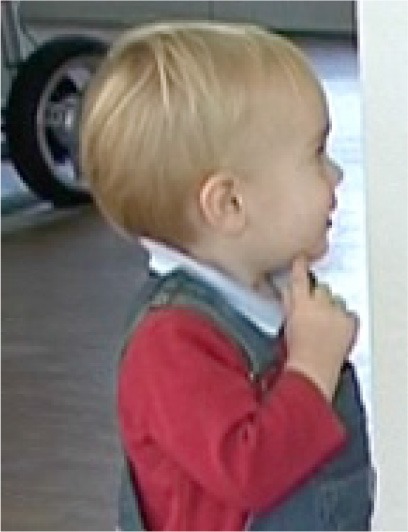			

Note that when pointing behind him, Ole's visual focus and the focus of his pointing diverge. Thus he orients toward two spaces at the same time: While maintaining eye contact with his mother, his pointing constitutes a domain of scrutiny in the opposite corner of the room. The mother formulates an assumption about the referent (line 22: “sandals”). By repeating the pointing and the local deictic (line 23), Ole indicates that his mother's assumption did not match what he wanted to convey and he prompts another attempt. The mother indeed produces another formulation (line 24) that he then accepts. Two issues are worth mentioning here: First, the example shows that Ole is able to create two diverging focuses of visual attention at the same time and thus to direct the coparticipant's gaze toward a distant space. Hence, he is able to initiate the first two jobs. The location of the target and the construal of the referent is left to the adult. Second, the episode provides an excellent example for our claim that “constituting a domain of scrutiny,” “locating a target,” and “construing the referent” are, in fact, different jobs. The mother's wrong assumption clearly shows that orienting toward a search space does not automatically imply the location and construal of the referent.

Table 2 summarizes the devices adults and children employ to constitute a domain of scrutiny.

**Table 2:** **Adults' and children's devices for constituting a domain of scrutiny**.

	**9–14 months**	**15–17 months**	**18–22 months**	**23–24 months**
Adult	Initiates job by setting up a conditional relevance for orienting toward the domain of scrutiny. Devices: Where question (prosodic emphasis on interrogative/search) or summons (“HERE”) Marking search space (book) by moving and lifting it Providing time for exploring the materiality of the book/Scrutinizing the search space Rendering general features of depictions visible Demonstrating the use of the book	Book-reading setting: Job is skipped as soon as the child understands the book as a potential domain of scrutiny		
Child	Responds by orienting toward and exploring the domain of scrutiny	Play setting: Initiates job by setting up a conditional relevance. Devices: Directing the adult's attention toward distant entities by establishing diverging focuses Pursuing a response/Reestablishing conditional relevancies		

#### Locating the target (Job 3)

##### 9–14 months

This job requires the recipient to determine a certain target in the domain of scrutiny. Again, this involves a perceptual effort. In interactions with very young children, adult coparticipants enhance the perceptibility of the act of locating. In Excerpt (14), the mother makes her own search both visible and audible.

**Table:** 

(14) 01-BR-dog (10 months)					
003	O:	((touches book with rH))			
		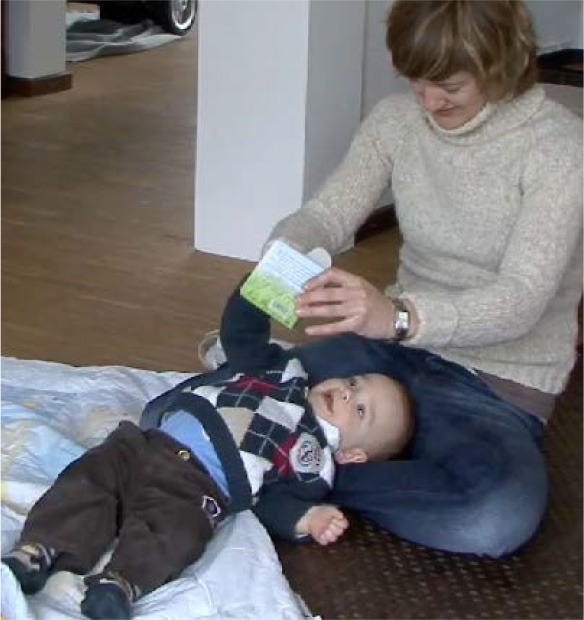			
**004**	**M:**	**°hhh;**			
**005**		**|bs::::t**,			**|**
		**|((moves IF over picture))**			**|**
006		|OH:::	eine MAUS;|		
			a MOUSE;		
		|((turns page)) |			
**007**		**|bs:::t**,			**|**
		**|((moves IF over picture))**			**|**
008		eine	KATze;		
		a	CAT;		

As soon as both participants share a visual focus on the domain of scrutiny (line 3), the mother sustains the child's attention by breathing in. She then overtly displays the search with her eyes by moving her index finger across the page until an object is found. Temporally aligned with the movement of the finger, she produces a lengthened sound (line 5 and again in line 7) that ends exactly at the moment when the object is located. In this way, the mother makes the relevant action—locating an object—observable. Her finger is being used to *guide* Ole's visual focus. By following the movement of the index finger, Ole can locate the object at exactly that moment when the end of the search is marked vocally (“bs:::t”). Immediately afterwards, the target is also labeled [line 6 and 8, see section Construing the Referent (Job 4)].

In this example, a perceptual action is carried out publically and observably (Kidwell and Zimmerman, 2006). This facilitates the child to coordinate her or his attention (Rader and Zukow-Goldring, 2010; Pitsch et al., 2014), enabling her or him not only to locate the target but also to perceive the coparticipant's perception. Given that not only mutual perception of an object but also reciprocal “perception of being perceived” (Hausendorf, 1995, p. 186) is a *sine qua non* for establishing reference (and interaction in general), this way of making perceptual acts observable for the coparticipant is particularly suited to acquaint the child with the reciprocal perception of being perceived.

Another device that adults employ is where questions. In the previous section (Job 2), we showed that where questions are used initially to evoke a searching stance in the child. As the interaction moves forward, the second implication of the question is made relevant, namely the request to locate *something in particular*. Analogous to the previous jobs, the job of locating a target becomes the subject of an adjacency pair. Forming the first pair part, the where question makes the action of locating (the second pair part) conditionally relevant. In this way, locating a target becomes part of the participants' obligations in a playful way.

**Table:** 

(15) 07-BR-spoon (9 months)						
004	M	|WO:	is	der	lÖffel;	|
		WHE:RE	is	the	spoon;	
		|((moves book, lifts it up))				|
**005**		**WO::**	**ist**	**der**	**lÖffel;**	
		**WHE::RE**	**is**	**the**	**spoon;**	
006	L	((touches book with face))				
007	M	WO	ist	der	lÖffel?	
		WHERE	is	the	spoon?	
008		[| < < breathy> DA:> ist der löffel.|]				
				THE:RE is the spoon.		
		|((points to spoon))				|
009	L	[((places rh on picture))]				
		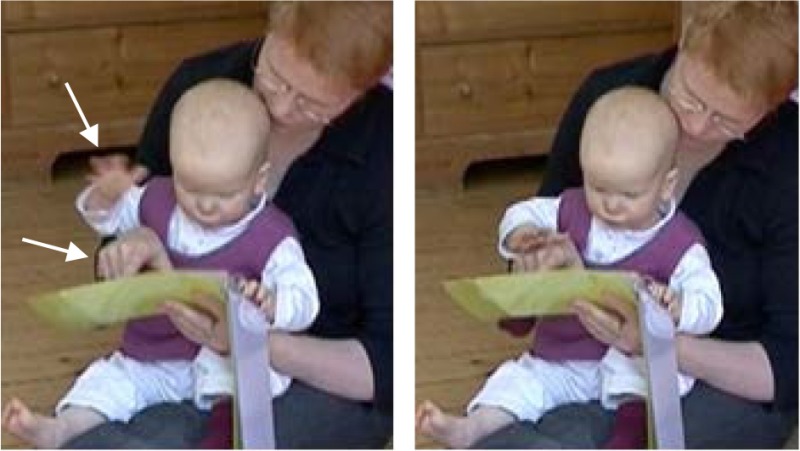				
010	L	[((lh touches picture, fingers splayed))]				
		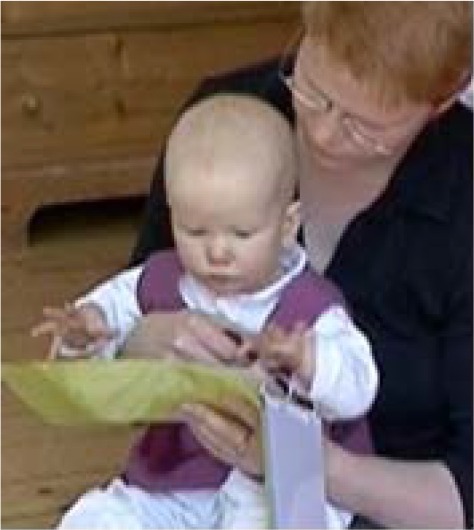				
011	M	[ < < ☺>DA:]	ist	der	LÖFfel-> =	
		THE:RE	is	the	spoon-	

In line 5, the conditional relevance is *reestablished*. Now Lea touches the book with her face (line 6). Reestablishing the conditional relevance again (line 7), the mother does not ratify Lea's action (touching the book with the face) as an adequate reply. Only now, when a response is observably absent, does the mother answer the question herself, thus taking over Lea's responsibility. In her turn, the mother temporally aligns the point with the local adverb “THE:RE,” which is not only accented but also produced with a breathy voice. Because “da/THE:RE” is emphasized repeatedly in this way, we refer to this device as the *emphatic da/there*. The emphatic da/there marks the fulfillment of the conditional relevance (i.e., the achievement of the goal of the search), and thus resolves the tension built up by the question (see Rossmanith et al., 2014).

In other words, crucial devices for locating the target—pointing and the verbal deictic—are again performed visibly and audibly and thus made available for the child. In concert with her mother, Lea brings her right hand to the book. Stopping the movement (line 9), she first observes the mother's pointing and then splays out her fingers before tapping the target. This movement is treated by the mother as a meaningful action. Using smile voice (Couper-Kuhlen and Barth-Weingarten, 2011), she both formulates and ratifies Lea's action (line 11). This way, she conventionalizes Lea's movement that now becomes a communicative means (Lock, 1980; Marcos, 1991).

Another device adults employ is *manual guiding:*

**Table:** 

(16) 07-BR-mug (11 months)						
019	(2.5)					
**020**	**M**	**|DA::**	**ist**	**der**	**becher;**	**|**
		**THE::RE**	**is**	**the**	**mug;**	
		**|((guides Lea‘s hand, [taps on picture))**				**|]**
		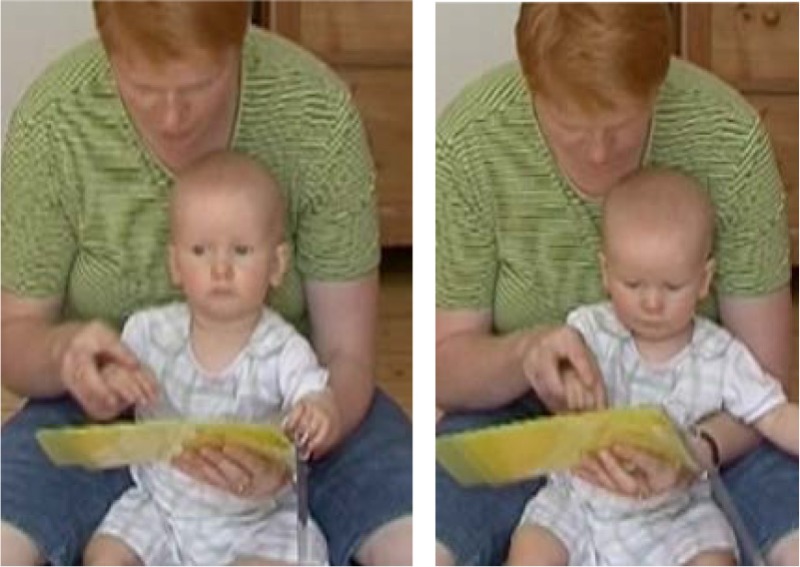				
021	L	[((looks at picture)) ]				
022	M	|DA:	ist	der	becher;	|
		THE:RE	is	the	mug;	
		|((taps on picture)) |				

Before locating the target verbally, the mother has taken Lea's right hand. Note that the mother's index is positioned on Lea's metacarpus and pushes the other fingers downwards. Overlapping with her verbal utterance, she then brings Lea's index finger closer to the book (line 20). The touch of the book induces Lea's visual attention (see Zukow-Goldring, 1996): She shifts her gaze to the book (line 21). As soon as Lea looks at the book, the pointing is repeated. Again, the emphatic da/there and the touch of the book are temporally aligned (line 20 and line 22). Hence, what is made available here is not only the movement and the local adverb but also the sequential position in which the action is expected.

##### 15–17 months

From 15 months onward, the children point without help. More importantly, they use this device in two different sequential positions, either as a *response* to the adult's where question or as an *initiative* to start off the job of locating. Pointing is now clearly established as a communicative device (Marcos et al., 2003). In extract (16), Lea responds to her mother's initiation.

**Table:** 

(17) 01-BR-lion (17 months)								
011	M	wo	ist	das	AUge,			
		where	is	the	EYE,			
**012**	**L**	**[|!DA:!;**					**|]**	
		**!THERE!;**						
		**[|((points to eye))**				**|]**		
		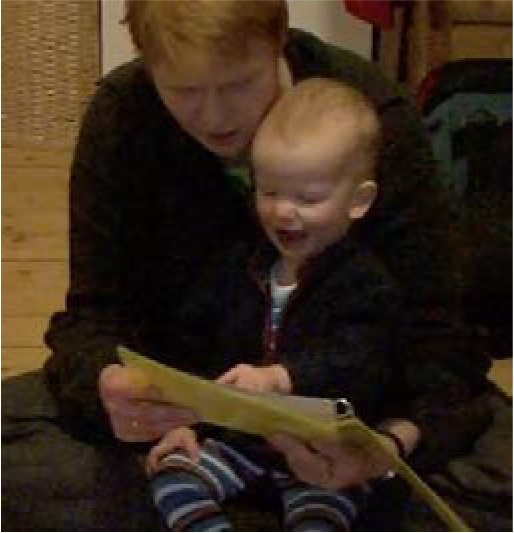						
013	M	[DA::]	is	das	AUge	vom		kleinen
		THE::RE	is	the	eye	of	the	little
014		löwen; =	genau;					
		lion;	exactly;					

Note that the prosodic design of the where question has been altered. The adult no longer places the focus accent on the interrogative pronoun but stresses the referent instead. This reflects heightened expectations regarding the child's understanding of the activity: The adult presupposes that the child has taken a searching stance and can now also focus on the object of the search.

The child responds to where questions by pointing and producing the verbal deictic “da”/“there.” The local adverb is temporally aligned with the point and produced with an extra strong accent (line 12). Hence, it closely resembles the mother's *emphatic da/there*. Because the referent is already mentioned in the adult's where question, locating the target and identifying the referent are achieved at once. Now that the child consistently produces the second pair part, the mother expands the sequence. She not only reformulates the child's utterance as a syntactically complete sentence (line 13) but also produces an evaluation (line: 14: “exactly;”), thereby transforming the adjacency pair into a three-part structure. This structure, known as IRE (Mehan, 1979: initiation, reply, evaluation), is typically observed in formal and informal learning contexts. The book-reading activity is thus turned into an instructional routine (Tarplee, 2010), casting the caregiver in the role of the instructor and the child in the role of the instructee.

This contextualization of the activity goes hand in hand with two other innovations: As soon as establishing reference is achieved smoothly, the adult heightens the demand by asking *series of questions* (see also Murphy, 1978). Furthermore, the adult *other-initiates self-corrections* (Schegloff et al., 1977) when the child's response is inaccurate. Excerpt (18) illustrates this finding.

**Table:** 

(18) 07-BR-peg (17 months)						
**005**		**LEa**	**wo**	**ist**	**der**	**TISCH**.
		**LEa**	**where**	**is**	**the**	**TABle**.
006	L	((points to table))				
		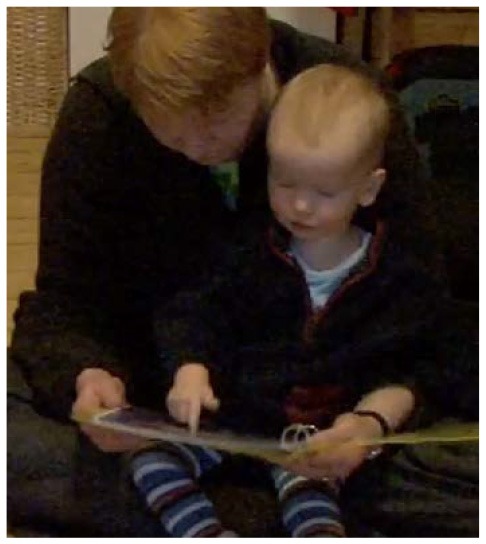				
**007**	**M**	**und**	**wo**	**ist**	**die**	**KLAMmer?**
		**and**	**where**	**is**	**the**	**PIN?**
008	L	((points to other part of the table))				
009		< < nodding> WUW;>				
**010**	**M**	**die**	**!WÄ!scheklammer;**			
		**the**	**!PIN!;**			
**011**		**zeig**	**mir**	**mal**	**die WÄscheklammer**.	
		**show**	**me**	**the**	**clothesPIN**.	
012	L	((points to pin))				
013	M	< < creaky> AH::> die wäscheklammer				
		the clothespin is				
		ist am TISCH-				
		on the TAble-				

After Lea has answered the first question (line 5), the adult immediately produces a second question (line 7) that asks for another detail. Withholding an evaluative receipt (Filipi, 2013) and repeating the request once more (line 10), the adult other-initiates a correction. Note that the request is also explicated (line 11 “show me”), thereby making it easier for Lea to understand that the activity has been halted, and that a revision of the previous utterance is expected. Lea indeed interprets this as a *request to self-correct* her response: She corrects her answer by pointing to another detail of the picture (line 12), and this is confirmed by the mother (line 13).

Between 15 and 17 months, the children in our study also began to start the job of locating:

**Table:** 

(19) 01-BR-stirring (16 months)		
001	O:	((turns page))
**002**		**|((points to spoon))|**
		**|mh::; |**
		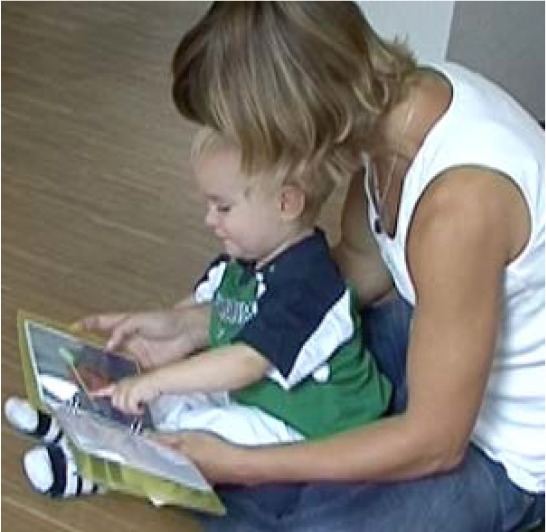

After turning the page, Ole immediately initiates the job of locating a target by vocalizing and pointing. At the same time, Ole produces a vocal gesture (line 2: “mh::;”) with which he labels the target [Job 4; we return to this gesture in the next section (Job 4)]. Hence, Ole has accomplished two jobs at once: he has located and identified the referent.

##### 18–22 months

From 18 months onward, children no longer display any difficulties in locating targets. In the book-reading routine, no further innovations could be observed with regard to the third job. New developments could be observed, however, when adults replaced their where questions with what questions, thereby requiring the child to label the referent her- or him self next section.

Table 3 summarizes the devices adults and children employ to locate the target.

**Table 3:** **Adults' and children's devices for locating a target**.

	**9–14 months**	**15–17 months**	**18–22 months**	**23–24 months**
Adult	Initiates job by setting up a conditional relevance for locating a target. Devices: Where questions (prosodic emphasis on target) Taking over the task of locating (in place of child) Demonstrating the action by making their own perception observable Manual guiding Distinguishing between “meaningful” and ”not meaningful” movements, formulating the child's action (temporally aligned)	Initiates job/responds to child's initiations Where questions in the context of three-part sequences → contextualizes activity as instruction Other-initiating self-correction		
Child	Responds by coordinating visual attention	Responds to/Initiates conditional relevance. Devices: Pointing Pointing + emphatic DA/THERE		

#### Construing the referent (Job 4)

##### 9–14 months

Although the younger children in our study do not yet possess the conventional communicative means to construe a referent, they are nonetheless being involved in this job. This is achieved by the adult's choice of a particular question format: Because the referent is already given in the where question, the act of locating coincides with construing the referent. When the child observably cannot not deal with this demand, the mother either assists by manual guiding or takes over the job, thus demonstrating how to deal with the interactive demand see previous section.

##### 15–17 months

From 15 months onward, the children in our study contributed to the job of construing the referent in a substantive way.

**Table:** 

(19) 01-BR-spoon (17 months)		
001	O	((turns page))
**002**		**|((points to mug in the book))|**
		**|pf::|**
003	M	°h:::;
**004**		**was ist DAS?** =
		**what is that**
005		[ = ne TASse ] mit EInem?
		a mug with a
006	O	[points to mug]
**007**		**|ÖFfel;|**
		**oon**
		**|((circular movement))|**
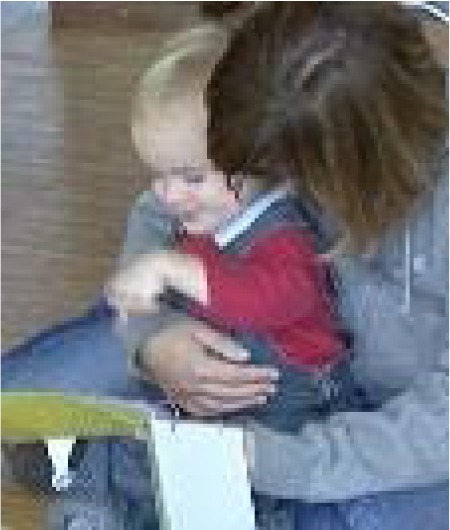		
008	M	LÖFfel;
		spoon
009		[geNAU::;]
		exactly
010	O	[((repeats circling movement))]

Ole initiates the job of locating a target and simultaneously depicts the movement and sound of drinking (line 2). Thus, he deploys a depictive practice that Streeck (2008, p. 295) terms *acting:* “the gestural action of the hand shows the practical action of a hand” and evokes an action. In this case, it is not the hand, but the mouth that represents itself in the action of drinking. With this depiction, Ole construes the referent. Now, the mother increases the interactive demand: She no longer uses where questions but asks *what questions* (line 4) that require the child to take on the main work of construing the referent (Murphy, 1978). Ole produces the verbal label “ÖFfel;” (line 7), which is aligned with a circling movement. The spoon is thus “indirectly represented by a schematic act that ‘goes with”’ it, a practice that Streeck (2008, p. 293) terms *handling*: “A motor schema or prehensile posture is coupled with an affordance of the referent.” Ole has “invented” this gesture (Behne et al., 2014) in previous episodes. When an object (e.g., the spoon) has been labeled, his mother often extended the sequence by asking “and what does one do with it?” Ole responded with a stirring movement that was taken up by his mother. In this context, however, he does not employ the movement to refer to the activity but to the object itself. He thus reuses semiotic resources with a new method of representation (see Heller and Rohlfing, 2015).

##### 18–22 months

In this period, the adults continued to ask what questions. The interactive demands for the child increased in two respects:

**Table:** 

(20) 07-BR-mug, hearts (22 months)			
011	M	was ham wa DA?	
		what do we have THERE?	
012	L	gε:	
013	M	SAG ma,	
		SAY,	
014		= was IS das?	
		what IS that?	
015	L	TASse.	
		MUG.	
016	M	ne TASse-	
		a MUG-	
017		= und WAS is obendrauf?	
		and WHAT is on top of it?	
018	L	|LÖFfel.	|
		SPOON.	
		|((points to picture))	|
020	M	|und was is das hier AUF der TASse?	|
		and what is that here ON the mug?	
		|((taps on picture))	|
021	L	pεtseɐ ;	
022	M	↑HERzen;	
		HEARTS;	
023	M	[der LÖFfel is auf der HERZtasse.	]
		the SPOON is on the HEART mug.	
024	L	[|((turns page))|	]
		|ja|	
		yes.	

When the child produces an unintelligible label (line 12), the adult systematically reestablishes and explicates the conditional relevance (lines 13–14). Halting the progression of the activity, the child is required to attend to the articulation of the word (line 15). In other cases, the adults reformulate the child's utterance, thus modeling the articulation of the word (line 22). Furthermore, the series of questions asking for familiar objects is extended (here: lines 17 and 20). The labels are then combined into one “thick description” (line 23).

##### 23–24 months

In the following months, the sequential pattern remained the same. Being ascribed the main responsibility for construing and labeling the referent, the child relied increasingly on verbal resources alone (see Murphy, 1978; Ninio and Bruner, 1978):

**Table:** 

(21) 07-BR-dino (24 months)		
001	L	|((oints to picture))|
		|DInoSAUrier;|
		DInoSAUR;
002	M	ja::,
		yes::,
003		und was ist oben AUF dem
		and what is there ON top
		dinoSAUrier?
		of the dinosaur?
004	L	LÖwe;
		LION;

Table 4 summarizes the devices adults and children employ to construe the referent.

**Table 4:** **Adults' and children's devices for construing the referent**.

	**9–14 months**	**15–17 months**	**18–22 months**	**23–24 months**
Adult	As long as where questions are asked, Jobs 3 and 4 merge For the devices, see Table 3	Initiates job by setting up a conditional relevance for labeling familiar objects What questions	Initiates job by setting up series of conditional relevancies for labeling. Devices: Reestablishing conditional relevance or initiating self-corrections Reformulating the child's utterance Asking series of questions	
Child	Fulfills conditional relevance. Device: Pointing	Fulfills conditional relevance. Devices: Acting gestures Handling gestures Pointing + verbal label	Fulfills conditional relevance and initiates job. Devices: For responding: pointing + verbal label Verbal label For initiating: what question	

### Longitudinal comparison: children's devices and shares in the jobs

In the following section, we track changes in the *children's* devices and shares in the jobs across time. The longitudinal comparison reveals changes in two areas: On the level of jobs, the children came to understand the mechanism of conditional relevancies. On the level of devices, the children first made use of non-verbal resources that were then combined with and partially replaced by verbal resources.

#### Developments on the level of jobs

As demonstrated above, establishing reference was achieved within four jobs that were each organized as an adjacency pair. Initially, each job was initiated by the adult who produced the first pair part. The children then increasingly displayed their understanding of the sequential implication by producing the second pair part. The age at which children started to orient toward conditional relevancies differed depending on the job: Whereas the conditional relevancies of establishing visual perception as a relevant resource (Job 1) were already responded to at 9 months of age (Excerpt 1), the implications of constituting a domain of scrutiny and locating a target first needed to be demonstrated by the adult. Only at the age of 15 months did the children produce conditionally relevant and conventional actions such as pointing to the target (Excerpts 13 and 17). Shortly afterwards, they also occasionally set up conditional relevancies for locating a target themselves (Excerpt 19). Whereas they started to initiate Jobs 1–3 by 15 months, we could observe initiations of construing the referent only at the age of 18 months (Excerpts 19 and 21).

In sum, on the level of jobs, the child's participation developed from being *responsive to conditional relevancies* to *proactively setting up conditional relevancies*. Furthermore, the children seemed to work their way forward through the sequential order: Both children mastered the initial jobs first before they occasionally began to initiate Job 4 and to oversee the whole sequential organization.

#### Developments on the level of devices

For the devices, the longitudinal comparison suggests that the children adopted means that had been used previously by the adult co-participant. At 15 months, the children initiated Job 1 by producing sharp intakes of breath and interjections (Excerpts 5 and 6); at the age of 24 months, they also employed what questions (Excerpt 8). All these devices had been used consistently by the adult. Likewise, the children acquired devices for locating a target that the adult co-participant used throughout the episodes: Pointing and pointing aligned with the emphatic da/there became a part of the children's repertoires around the age of 15 months (Excerpt 17). With respect to the fourth job, the children were first expected to identify a referent by pointing. When the mothers increased the demand by asking what questions instead of where questions, the children started to use depictive gestures (Excerpt 19). Remarkably, the use of the gestures was not based on imitation; instead, their “invention” (see Behne et al., 2014) had been “provoked” by the adults' questions about depicted objects such as “What does one do with a spoon?” (Heller and Rohlfing, 2015). Depictive gestures were replaced increasingly by verbal means (aligned with pointing) at the age of 18 months (Excerpt 20). This is in line with findings reported by Capirci et al. (1996), Goldin-Meadow and Butcher (2003), and Mai-Rong et al. (2015).

Hence, on the level of devices, development proceeds from using *somatic and non-verbal resources* to using *verbal and symbolic resources*. However, somatic and non-verbal resources remain important across development and continue to facilitate the smooth execution of the jobs. The use of somatic and non-verbal resources allows children to actively participate in establishing reference long before they are able to speak. From 15 months onward, the sequential machinery of establishing reference runs smoothly. An important finding is, then, that at this age, children have acquired essential competencies for establishing reference even if they do not have the verbal resources at their command.

The longitudinal comparison shows that at an early age, the children's shares in the jobs do not conform to what is usually expected from competent participants in establishing reference (see Mehus, 2011, for a similar finding). Nevertheless, all jobs are accomplished. When the child does not respond to conditional relevancies in the expected way, the adult takes over the child's tasks and does “extra work” (see Hausendorf and Quasthoff, 2005). We shall pursue this aspect in the next section.

### Longitudinal comparison: adults' devices and shares in the jobs

In the following section, we track changes in the *adults'* devices and shares in the jobs. On the level of jobs, the adult provided support for the child to understand the mechanisms of conditional relevancies. On the level of devices, the adult increasingly replaced somatic resources by symbolic ones and also required the child to employ verbal means.

#### Changes on the level of jobs

*Setting up conditional relevancies* is the basis for initiating the jobs. In interactions with young children, this was done consistently by the adult (Excerpts 1–4). Furthermore, the adult made sure that the conditional relevancies remained in force when they were not responded to adequately by:

- reestablishing and sometimes also explicating sequential implications;
- assisting the child in producing the second pair part of an adjacency pair;
- taking over the task of producing the relevant next action when the child did not manage to produce the expected response.

These supportive practices ensured the maintenance of the sequential order. Their use underwent considerable changes over the course of the child's second year of life:

*Reestablishing conditional relevancies* was observed throughout the child's second year. At the beginning, adults reestablished conditional relevancies when a response was absent (Excerpt 15). In this way, they ensured that the sequential implication remained in force (see Filipi, 2013: “pursuing a response”; Hausendorf and Quasthoff, 2005). Later, conditional relevancies were also reestablished when the response was inadequate; for example, when the child located the wrong target or produced an unintelligible label (Excerpt 20). This prompted the child to correct the response (see Tarplee, 2010). Explications of sequential implications (e.g., “show me” or “say, what is that”; see Hausendorf and Quasthoff, 2005) could be observed only from 17 months onward (Excerpts 18 and 20) when the child displayed sufficient understanding of verbal utterances. Before this, the caretakers tended to rely on making sequential implications perceptible (see below).

*Assisting* the child in producing a second pair part is contingent on establishing a conditional relevance. This was used mainly to get perceptual tasks done. Between 9 and14 months, the adult assisted the child in locating a target by guiding her or his visual focus and manual guiding (Excerpts 14 and 16). As soon as the children were able to locate a target themselves, assistance was omitted. These observations extend previous findings reported by Zukow-Goldring (1996) showing that the child's attention is “educated.” Our analyses show that this “education” also includes the sequential position in which the action is expected.

*Taking over* a task, that is, producing the second pair part in place of the child, was realized only when a response remained absent even after reestablishing a conditional relevance (Excerpt 15). This is consistent with findings reported by Hausendorf and Quasthoff (2005). As soon as the child displayed her or his ability to produce the expected second pair part, the adult refrained from taking over the child's task. Taking over thus served two functions: First, it guaranteed that the job was in any way accomplished at all and that the activity could continue; second, it made the expected action observable for the child and provided a model for what to do when and how.

With the four practices of (1) setting up conditional relevancies, (2) reestablishing and explicating conditional relevancies, (3) assisting the child in producing a second pair part, and (4) taking over a task, the adults ensured that the jobs were being accomplished no matter how much the child was able to contribute. Thus, they were oriented toward the successful achievement of reference. At the same time, the highly differentiated employment of the four practices was oriented toward gradually reducing the adult's “extra work” (Hausendorf and Quasthoff, 2005) and arriving at equal contributions to establishing reference.

As soon as the children mastered certain jobs, they were also given the opportunity to set up conditional relevancies themselves. This observation is consistent with what Bruner describes as “handover” (Bruner, 1983, p. 60). In addition, our analyses revealed that the focus of the conditional relevancies shifted *from perceptual to semantic* ones. In interactions with young children, adults focused on those jobs that mainly entailed perceptual demands. The use of where questions in the first half of the second year made it easier for the dyad to achieve joint reference. Because the referent was already given with the adult's where question, the jobs of locating the target and construing the referent merged together and could both be achieved by pointing. Around 17 months, where questions were replaced consistently by what questions. This shifted the focus to the semantic task of construing and labeling the referent (Excerpts 19 and 20; see Miller and Weissenborn, 1979, for a similar finding). This also made it possible to differentiate familiar referents from unfamiliar ones (see Bruner, 1976) and thus to direct the child's attention to “new objects.”

#### Changes on the level of devices

On the level of devices, it could be observed that sequential implications were first made understandable by *sensorily perceptible* means (see Zukow-Goldring, 1996) and increasingly by *symbolic* (linguistic) means. This could be seen in the design of the what questions. At 9–12 months, mothers made their own excitement perceptible by prefacing what questions with a sharp intake of breath or an interjection (Excerpt 3). At 20 months, these prefaces were usually omitted (Excerpt 20). Likewise, the design of the where questions changed over time. At 9 months, the mothers conveyed the expectation of searching as such by stressing and lengthening the interrogative and additionally lifting the book (Excerpt 14). Eight months later, the expectation became more specific when the target of the search was emphasized (Excerpt 16). It could be observed that the shift from perceptual to semantic tasks went hand in hand with the expectation that the child should increasingly use verbal resources (see Ninio and Bruner, 1978; Bruner, 1983). Whereas conventional non-verbal means such as pointing or gestural depiction continued to be important resources for establishing reference, the adult also asked the children to use verbal means.

In sum, the longitudinal analysis reveals that the availability of devices on the side of the children and their growing shares in the jobs correspond to changes in how adults maintain the sequential order of establishing reference by making use of the supportive practices described above. So far, we have shown that these practices ensured the accomplishment of reference between unequally competent partners in the here and now of each particular episode, and we have shown how this was done. In the next section, we ask what interactive mechanisms these practices are based on and how they drive the acquisition of reference.

## Discussion: what are the driving forces in the acquisition of reference?

On the basis of video-recorded labeling interactions of shared book reading and free play involving children from the age of 9–24 months and their mothers, we sequentially analyzed how the participants dealt with perceptual and semantic ambiguities and eventually established stable patterns of bodily, perceptual, and interactive coordination. In the subsequent longitudinal analysis, we tracked changes in the children's and adults' behavior and examined how caregivers managed to involve children in establishing reference.

Starting from the assumption that reference is fundamentally an interactive achievement, we proposed a descriptive instrument that rests upon empirically reconstructed jobs: (1) establishing visual perception as a relevant resource (2) constituting a domain of scrutiny, (3) locating a target, and (4) construing the referent. Differentiating between jobs and devices allowed us to relate differences in the children's participation in establishing reference to the adults' practices of sustaining the sequential organization.

Concerning the *devices*, our results (summarized in Tables 1–4) indicate that children adopted means that had been used previously by the adult. Importantly, Vygotsky (1998) point out that children can pick up only those means that are within their zone of proximal development. Our analyses demonstrate how caregivers fine-tuned their communicative expectations by making sequential implications understandable first by sensorily perceptible and only later by symbolic means. This progression was mirrored in the child's behavior proceeding from using somatic and non-verbal to using verbal and symbolic resources. Importantly however, it was the use of somatic resources that allowed the child to participate actively in establishing reference. These resources continued to facilitate the smooth execution of the jobs.

With regard to the level of *jobs*, our analyses extend previous findings in which only two tasks (i.e., getting and maintaining attention) were assumed to be involved in establishing reference (Estigarribia and Clark, 2007). Our sequential analyses of dyadic book reading and free play showed that, in fact, establishing reference involves four tasks. Analyses of misunderstandings further demonstrated that the jobs “locating a target” and “construing a referent” are indeed two different jobs that entail perceptual demands for the former and semantic demands for the latter. Furthermore, we showed that each of the four constitutive jobs of establishing reference is organized as an adjacency pair. Thus, each job requires contributions from both participants, with one participant setting up a conditional relevance and the other partner producing the expected second pair part. Joint reference is established successfully when each of the four consecutive relevancies is fulfilled. The four jobs constitute the pragmatic frame (Rohlfing et al., 2016) of establishing reference in which the sequential order of actions and the devices for realizing them become accessible in their pragmatic functions.

It could be observed that the adults employed supportive practices such as *setting up, reestablishing*, and *explicating* conditional relevancies; *assisting* the child; or *taking over* the child's task in order to maintain the sequential order. In the remainder of this article, we shall argue that these practices work as a driving force in the acquisition of reference, because they make use of basic features of interaction: *conditional relevancies, recipient design*, and *observability*. Our analyses show that these features are specifically contextualized in interactions between unequally competent partners (Wootton, 1997; Hausendorf and Quasthoff, 2005).

From an acquisitional perspective, the *conditional relevancies* (Schegloff and Sacks, 1973) that initiate each of the four constitutive jobs can be understood as *interactive demands* (Hausendorf and Quasthoff, 2005, p. 270). In constraining the child's actions, the adult's interactive demands serve as a scaffold (Bruner, 1978, p. 19) or yardstick for the child to act in expected and coordinated ways. The more competent partner supports this process by differentiating between acceptable and inacceptable responses (Bruner, 1983; Mehus, 2011). In this way, the child increasingly comes to draw on conventionalized resources (Lock, 1980).

Our longitudinal comparison revealed that the adults' interactive demands change considerably over time. They adapt or “design” their actions specifically “for” their recipients who display different degrees of competence. From a conversation analytic perspective, *recipient design* represents a constituent feature of interaction in general (Sacks, 1995). From a developmental perspective, *fine-tuning* (Bruner, 1983; Snow, 1995) can be understood as a form of recipient design. Changes in question designs provide ample evidence for the adult's *fine-tuning* (Bruner, 1983; Snow, 1995) to the child's developing competence. Likewise, the shift from where questions to what questions exemplifies how adults first reduce and then raise interactive demands. Our findings thus lend further support to the acquisitional effectiveness of the caregiver's dynamic adaptation to the child's abilities (Marcos, 1991; Snow, 1995; Zukow-Goldring, 1996; Wootton, 1997; Vygotsky, 1998; Hausendorf and Quasthoff, 2005; Forrester, 2013; Trueswell et al., 2016). In line with Vygotsky's zone of proximal development (Vygotsky, 1998), our findings suggest that the adults' support in fact enables children to come to grips with the sequential organization of establishing reference and to eventually initiate jobs by themselves.

Adults also make particular use of the *observability* of communicative actions (Goffman, 1967; Sacks, 1980). With this term we refer to the “systematic ways in which objects and people come to be available to others for inspection via their public character” (Kidwell and Zimmerman, 2007, p. 593; see also Kidwell and Zimmerman, 2006). Our analysis of interactions with not yet fully competent participants demonstrates that observability is *enhanced* with respect to three domains: the sequential structure, interactive expectations, and devices. First, adults increase the *observability of their own devices* by embodying their excitement or performing their location of a target both visibly and audibly. Our finding that those devices that were made particularly salient were then later used by the child, supports the claim that the enhanced observability of devices facilitates their acquisition by the child. Second, in their reactions to the child's responses, adults display whether and to what extent that response meets or fails to meet certain expectations (either confirming it, other-initiating corrections, or reformulating it). This observability of expectations helps the child to meet sequential demands and to gradually employ conventional resources. Finally, the observability of the sequential organization is increased through the book-reading routine itself: Its repetitive structure with several cycles of establishing reference helps the child to recognize the overall sequential scheme (Ninio and Bruner, 1978; Snow and Goldfield, 1983; Rohlfing et al., 2015) or “action arc” (Rossmanith et al., 2014, p. 8) of book reading in which each turning of the page marks the beginning of a new referential cycle.

Enhancing the observability of devices, expectations, and the sequential order can be conceived as a way of increasing the perception of the task structure—an idea that is also reflected in research on “referential transparency” (Zukow-Goldring, 1996; Rader and Zukow-Goldring, 2010; Trueswell et al., 2016; Yu and Smith, 2016). This research has mainly stressed the role of transparency for identifying the referent. Widening the lens on the whole process of establishing reference, our analyses reveal that the importance of transparency or observability also extends to devices for establishing reference and to the sequential organization as a whole.

In sum, we characterize the process of establishing references as a sequential order that is sustained by supportive adults. We conclude that the adults' supportive practices exploit basic features of interaction (conditional relevancies, recipient design, observability) that are specifically contextualized in interactions with less competent partners. Social interaction itself thus proves to be an important source of the child's communicative and cognitive development (Vygotsky, 1998; Hausendorf and Quasthoff, 2005). Further research should examine whether these supportive practices are realized intuitively by all caregivers. To fully answer this question, we need to investigate cases in which caregivers and children display difficulties in establishing joint reference. If caregivers barely establish and maintain the sequential organization described above, it could well be that the children in their care show delays in the acquisition of reference.

## Ethics statement

This study was carried out in accordance with the recommendations of Ethik-Kommission der Ärztekammer Westfalen-Lippe and the Medizinische Fakultät der Westfälischen Wilhelms-Universittät Münster with written informed consent from all subjects. All subjects gave written informed consent in accordance with the Declaration of Helsinki. The protocoll was approved by Ethik-Kommission der Ärztekammer Westfalen-Lippe and the Medizinische Fakultät der Westfälischen Wilhelms-Universität Münster.

## Author contributions

VH developed the descriptive instrument; KR collected the data; VH and KR analyzed the data and wrote the paper.

## Funding

This work was funded by the Deutsche Forschungsgemeinschaft as part of the CRC 673 “Alignment in Communication” at the Cluster of Excellence Cognitive InteractionTechnology “CITEC” (EXC277).

### Conflict of interest statement

The authors declare that the research was conducted in the absence of any commercial or financial relationships that could be construed as a potential conflict of interest.
