# Supplementary material for: Reference as an Interactive Achievement: Sequential and Longitudinal Analyses of Labeling Interactions in Shared Book Reading and Free Play
Source: Front Psychol. 2017 Feb 14;8:139. doi: 10.3389/fpsyg.2017.00139 (PMC5306378; doi:10.3389/fpsyg.2017.00139)
Supplement: Supplementary file 1 [file DataSheet1.docx]

## Appendix: Transcription conventions (GAT 2, Couper-Kuhlen and Barth-Weingarten, 2011)

*Sequential structure*

[ ] overlap and simultaneous talk

[ ]

= fast, immediate continuation with a new turn or segment

*Pauses*

(.) micro pause, estimated, up to 0.2 s duration appr.

(-), (--), (---) estimated pause of appr. 0.2–0.5, 0.5–0.8, 0.8–1.0 s duration

(0.5)/(2.0) measured pause of appr. 0.5/2.0 s duration

*Accentuation and segmental conventions*

SYLlable focus accent

!SYL!lable extra strong accent

:, ::, ::: lengthening, by about 0.2–0.5 s, 0.5–0.8 s, 08–1.0 s

*Final pitch movements of intonation phrases*

? rising to high

, rising to mid

– level

; falling to mid

. falling to low

*Loudness und tempo changes, with scope*

<<f> > forte, loud

<<p> > piano, soft

<<breathy> > change in voice quality as stated

*Other conventions*

°h / h° in- / outbreaths of appr. 0.2–0.5 s duration

°hh / hh° in- / outbreaths of appr. 0.5–0.8 s duration

| | simultaneous verbal and nonverbal activities

lh / rh left hand/ right hand

↑ / ↓ smaller pitch upstep / downstep
